# Centrifugation-Induced Stable Colloidal Silver Nanoparticle Aggregates for Reproducible Surface-Enhanced Raman Scattering Detection

**DOI:** 10.3390/bios15050298

**Published:** 2025-05-08

**Authors:** Tianyu Zhou, Zhiyang Zhang

**Affiliations:** 1 CAS Key Laboratory of Coastal Environmental Processes and Ecological Remediation, Yantai Institute of Coastal Zone Research, Chinese Academy of Sciences, Yantai 264003, China; zhoutianyu22@mails.ucas.ac.cn; 2 University of Chinese Academy of Sciences, Beijing 100049, China; 3 Center for Ocean Mega-Science, Chinese Academy of Sciences, Qingdao 266071, China

**Keywords:** surface-enhanced Raman scattering (SERS), colloidal silver nanoaggregates, centrifugation, β-cyclodextrin, signal reproducibility

## Abstract

Colloidal noble metal nanoparticle aggregates have demonstrated significant advantages in surface-enhanced Raman scattering (SERS) analysis, particularly for online detection, due to their excellent optical properties, spatial homogeneity, and fluidic compatibility. However, conventional chemically induced aggregation methods (such as salt-induced nanoparticle aggregation) suffer from uncontrolled aggregation, limited stability, and narrow detection windows, which restrict their quantitative and long-term applications. In this study, we developed a non-chemical method for fabricating stable colloidal aggregates from uniform β-cyclodextrin-stabilized silver nanoparticles (β-CD@AgNPs) via centrifugation. By precisely controlling the addition rate of silver nitrate, we synthesized β-cyclodextrin-stabilized silver nanoparticles with a uniform size. Surprisingly, these nanoparticles can form highly dispersed and homogeneous colloidal aggregates simply via centrifugation, which is completely different from the behavior of traditional ligand-modified nanoparticles. Notably, the resulting aggregates exhibit excellent SERS enhancement, enabling the sensitive detection of various dyes at nanomolar levels. Furthermore, they maintain a stable SERS signal (RSD = 6.99%) over a detection window exceeding 1 h, markedly improving signal stability and reproducibility compared with salt-induced aggregates. Additionally, using pyocyanin as a model analyte, we evaluated the quantitative performance of these aggregates (LOD = 0.2 nM), achieving satisfactory recovery (82–117%) in spiked samples of drinking water, lake water, and tap water. This study provides a facile strategy for fabricating stable colloidal SERS substrates and paves the way for the advancement of SERS applications in analytical sciences.

## 1. Introduction

Surface-enhanced Raman scattering (SERS) spectroscopy, with its capability for single-molecule detection and fingerprint identification, has become an indispensable tool in modern analytical science, being widely used in biochemical analysis, food testing, reaction process analysis, and other fields [1,2,3,4,5,6]. As a core component of SERS technology, the performance of the SERS substrate directly determines the sensitivity, reproducibility, and practicality of the detection system [7]. Based on the substrate morphology, existing SERS substrates can be divided into solid and colloidal types [2,8]. Solid substrates are typically prepared either via direct deposition (e.g., physical vapor deposition and electrochemical deposition) or by assembling colloidal nanoparticles onto a two-dimensional flat substrate [9,10,11]. Although the solid SERS substrates possess fixed nanostructures with stable SERS hotspots, the common methods (such as drop-casting) to prepare solid substrate usually lead to signal fluctuations and low reproducibility [2,12]. Furthermore, the development of uniform solid SERS substrates usually requires high equipment costs or complex experimental procedures [13].

In contrast, colloidal noble metal nanoparticle aggregates used as SERS substrates have attracted considerable attention due to their spatial homogeneity, mobility, and uniform signal distribution [14]. They are especially advantageous for detecting analytes that are prone to oxidation and volatilization. Under the influence of external chemical agents (such as organic ligands [15,16] or high-concentration salts [17]), noble metal nanoparticles in solution aggregate to form numerous dynamic “hotspots”. These three-dimensional SERS hotspots not only enhance detection sensitivity but also provide excellent optical uniformity [18]. Currently, the simplest and most effective method to achieve strong plasmonic enhancement in colloidal systems is to induce nanoparticle aggregation using inorganic salts [19]. The stability of noble metal nanoparticles in solution primarily relies on the electrostatic repulsion provided by the ions or ligands adsorbed on their surfaces [20]. When high concentrations of inorganic salts are introduced, the double-layer structure on the nanoparticles’ surface is disrupted, and electrostatic repulsion is significantly reduced, leading to aggregation driven by van der Waals forces [2]. During aggregation, numerous SERS hotspots form around nanoparticle contact points, significantly enhancing the Raman signal of analytes. However, chemically induced nanoparticle aggregation is typically uncontrollable, and excessive aggregation may lead to precipitation, which greatly reduces the temporal stability and reproducibility of the SERS signal, thereby shortening the detection window and limiting its potential in on-site detection and quantitative analysis. Furthermore, residual chemical inducers (such as salts or organic molecules) and their adsorption on the nanoparticles may interfere with the direct adsorption of analytes, further restricting the applicability of such substrates for detecting analytes with low adsorption affinity [21,22].

**Figure 1:**
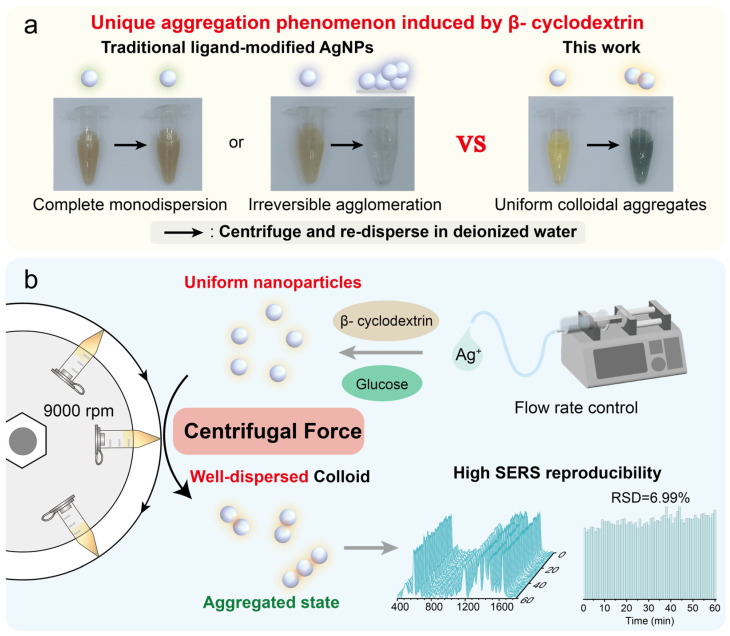
(**a**) Unique aggregation phenomenon induced by β-cyclodextrin. (**b**) Schematic illustration of centrifugation-induced highly dispersed silver nanoaggregates (AgNAs) for reproducible SERS detection.

To address this issue, we present a physical method for fabricating stable colloidal aggregates of silver nanoparticles via centrifugation-induced aggregation of uniform β-cyclodextrin-stabilized silver nanoparticles (β-CD@AgNPs). By precisely controlling the silver nitrate addition rate during synthesis, we obtained uniformly sized β-CD@AgNPs. Surprisingly, these nanoparticles readily self-assemble into uniformly dispersed colloidal aggregates through simple centrifugation. This is completely different from the behavior of various traditional ligand-modified silver nanoparticles after centrifugation. Notably, compared with salt-induced aggregates, the resulting colloids exhibit far superior stability at room temperature, leading to higher reproducibility of SERS signals. In addition, they demonstrate excellent SERS enhancement, enabling the detection of various dye molecules at nanomolar levels. When combined with microfluidic and automated sampling technology, this method facilitates continuous online high-throughput analyte analysis, significantly increasing analytical speed and efficiency. Using pyocyanin as a target analyte, the method achieved a detection limit as low as 0.2 nM and yielded satisfactory recovery rates in spiked samples of drinking water, tap water, and lake water. This approach offers a facile strategy for the rapid synthesis of highly stable, colloidal nanoaggregate-based SERS substrates, significantly enhancing SERS detection reproducibility and paving the way for advanced SERS applications in analytical chemistry (Figure 1).

## 2. Materials and Methods

### 2.1. Reagents and Materials

All reagents in this work were analytically pure. Chloroauric acid (HAuCl_4_·4H_2_O), sodium citrate (Na_3_C_6_H_5_O_7_·2H_2_O), silver nitrate (AgNO_3_), L-ascorbic acid (L-AA), glucose, Ethanol (EA), and sodium chloride (NaCl) were purchased from Sinopharm Chemical Reagent Co., Ltd. (Shanghai, China). β-cyclodextrin (β-CD) and pyocyanin were purchased from Aladdin Reagents (Shanghai, China). All solutions were prepared using sterilized ultrapure Millipore water (18.2 MΩ/cm). Crystal Violet (CV), Nile Blue A (NB), and Malachite Green (MG) were purchased from Shanghai Macklin Biochemical Technology Co., Ltd. (Shanghai, China).

### 2.2. Instruments

The micromorphology of NPs was observed using an S-4800 field emission scanning electron microscope (SEM) (Hitachi, Tokyo, Japan). TEM images were obtained using the Talos F200X G2 (Thermo Fisher Scientific, Waltham, MA, USA) at an acceleration voltage of 200 kV, and energy dispersive X-ray spectroscopy (EDS) was measured using a Super-X EDS system. UV-Vis absorption spectra were recorded from a Thermo Scientific NanoDrop 2000/2000C spectrophotometer (Thermo Scientific, Waltham, MA, USA). All the SERS spectra were measured using a Renishaw inVia Raman microscope (Renishaw, Gloucestershire, UK), with a 5× objective lens used for amplification and a 633 nm laser used as the excitation light source.

### 2.3. Synthesis of β-CD@AgNPs

By improving the method previously reported by Pan et al. [12], β-CD-modified silver nanoparticles (β-CD@AgNPs) were synthesized as follows: 15 mL glucose aqueous solution (0.013 M), 15 mL NaOH aqueous solution (0.01 M), and 30 mL β-CD (0.015 M) solution were mixed and heated at a constant rate. When the solution reached 60 °C, the AgNO_3_ solution (0.01 M) was infused at 0.8 mL/min (13 µL/min) using a syringe pump while maintaining vigorous stirring (400 rpm), ensuring homogeneous precursor distribution during nucleation. After the reaction was complete, the solution was cooled to room temperature.

### 2.4. Preparation of Centrifuge-Induced Aggregates

First, 1 mL of the β-CD@AgNPs solution, cooled to room temperature, was transferred into a 1.5 mL centrifuge tube. The centrifugation was set at 15 °C with a speed of 9000 rpm for 15 min. After centrifugation, 995 µL of the supernatant was removed and dispersed in 100 µL of deionized water.

### 2.5. SERS Measurements

A total of 90 µL of the solution containing different analytes was mixed with 10 µL of the centrifuge-induced aggregates and thoroughly blended. The mixture was then drawn into a capillary tube with an inner diameter of 0.9–1.1 mm and focused under a 5× objective lens of a confocal Raman microscope for testing.

### 2.6. FDTD Simulation

In order to quantitatively explore the electromagnetic field distributions of silver nanoparticle dimers under various aggregation states, we employed three-dimensional finite-difference time-domain (FDTD) simulations with Lumerical FDTD Solutions. A plane wave source of 633 nm wavelength, polarized along the *x*-axis, was introduced along the *z*-axis to illuminate the simulation domain. Perfectly matched layer (PML) boundary conditions were set on all boundaries to minimize unphysical reflections. Additionally, to capture the near-field gradients accurately, a refined mesh overlay of approximately 0.5 nm was applied in the vicinity of the nanoparticles. In these simulations, one silver nanosphere was fixed at 40 nm in diameter, while the second sphere was set to diameters of 40 nm, 25 nm, and 10 nm, each placed 2 nm apart to represent the interparticle gap. The complex refractive index of silver was taken from the CRC database to ensure accurate material dispersion properties. The simulation medium was assumed to be water (refractive index = 1.3), and the total simulation region was adjusted so that the boundary conditions would not interfere with the near-field region around the dimers.

## 3. Results

### 3.1. Synthesis of Uniform β-CD Stabilized AgNPs

Achieving uniform size and shape of metal nanoparticles is crucial for SERS applications as well as for other fields, such as biomedicine, catalysis, and electronics. However, synthesizing silver nanoparticles with a uniform morphology is a complex process due to various influencing factors [23]. In this study, we synthesized silver nanoparticles by modifying the method reported by Pan et al. [12], using glucose as a reducing agent and β-CD as a stabilizer. In contrast to Pan’s method, we employed a syringe pump to precisely control the silver nitrate addition rate, thereby investigating the effect of the addition rate on the morphology and dispersity of silver nanoparticles.

**Figure 2:**
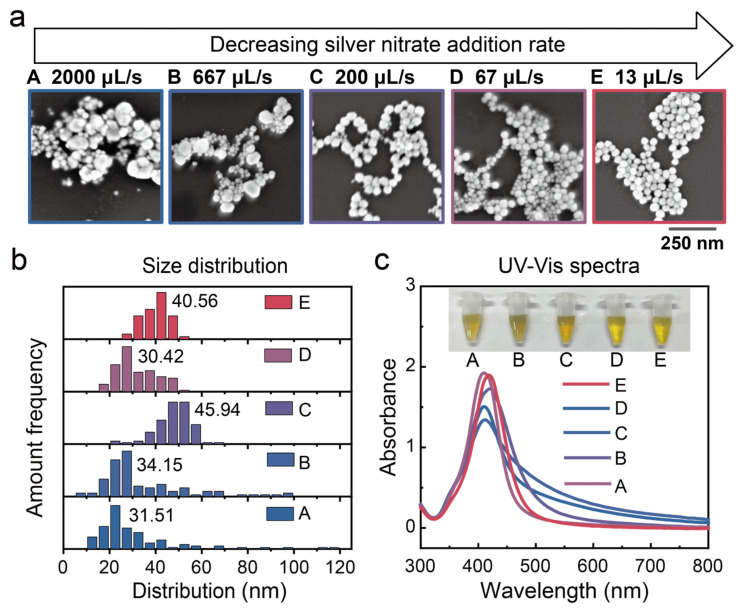
(**a**) SEM images of AgNPs synthesized at different silver nitrate dripping rates. (**b**) Corresponding size distributions of the AgNPs. (**c**) Solution colors and UV-Vis absorption spectra of different AgNP sols under monodispersed conditions.

Figure 2a presents scanning electron microscopy (SEM) images of β-CD@AgNPs synthesized under different silver nitrate addition rates. It is evident that when the addition rate is 2000 µL/s, the nanoparticles exhibit a highly non-uniform size distribution. As the addition rate decreases, particle size uniformity improves markedly, and the silver nanoparticles exhibit high homogeneity when the rate is reduced to 13 µL/s. Statistical analysis of the SEM images further confirms that lower silver nitrate addition rates yield nanoparticles with a more uniform, near-spherical morphology and a narrower size distribution (Figure 2b). Additionally, all colloidal samples display a distinct localized surface plasmon resonance (LSPR) peak around 410 nm in the UV-Vis absorption spectrum, indicating the successful synthesis of monodisperse silver nanoparticles (Figure 2c). Moreover, the plasmon bands narrow as the addition rate decreases, further indicating a significant improvement in particle size uniformity.

We propose that this phenomenon can be attributed to two key factors. First, a lower precursor addition rate helps maintain a balanced silver nitrate-to-glucose ratio and reduces supersaturation during nucleation. This results in a consistently low nucleation rate, favoring the formation of larger and more uniform crystals. Second, the adsorption of β-CD plays a crucial role in stabilizing the silver nanoparticles and controlling their morphology. A slower addition rate allows sufficient time for β-CD molecules to adsorb onto the freshly formed nanoparticle surfaces and stabilize them, thereby preventing the uncontrolled reduction of silver ions and subsequent crystal growth. Together, these two factors contribute to the formation of uniformly shaped and well-defined silver nanoparticles. In contrast, rapid injection of silver nitrate results in a spatially inhomogeneous silver nitrate-to-glucose ratio, leading to significant variations in nucleation conditions. Moreover, the adsorption of β-CD on silver crystal surfaces does not reach equilibrium, thereby compromising shape control. As a result, the synthesized nanoparticles exhibit pronounced size polydispersity, as indicated by the broad LSPR peak observed in the UV-Vis absorption spectrum. These findings offer valuable theoretical insights into the controlled synthesis of uniform silver nanoparticles and highlight unique phenomena observed during subsequent centrifugation.

### 3.2. Centrifugation-Induced Aggregates of β-CD Stabilized AgNPs

Centrifugation is an essential step in the synthesis and post-processing of noble metal nanoparticles [24]. Under appropriate chemical conditions and centrifugation parameters, the aggregation state of nanoparticles typically remains unchanged (Figure S1a). However, in the presence of weak ligand protection, excessive centrifugation speed, high salinity, or improper pH conditions, nanoparticle aggregation may occur during centrifugation. This undesirable phenomenon leads to irreversible adsorption, aggregation, and sintering of nanoparticles on the tube walls, at the tube bottom, or between nanoparticles, which can adversely affect their concentration, monodispersity, and subsequent surface modifications. For instance, citrate-reduced gold nanoparticles tend to aggregate on tube walls under weakly acidic conditions (Figure S1e), resulting in red-shifted and altered peak shapes in the LSPR spectrum. Similarly, in high-salinity conditions, citrate-reduced silver nanoparticles irreversibly aggregate on tube walls during centrifugation (Figure S1d). Moreover, excessive centrifugation speed can cause severe aggregation at the tube bottom, making redispersion difficult without ultrasonication (Figure S1c).

**Figure 3:**
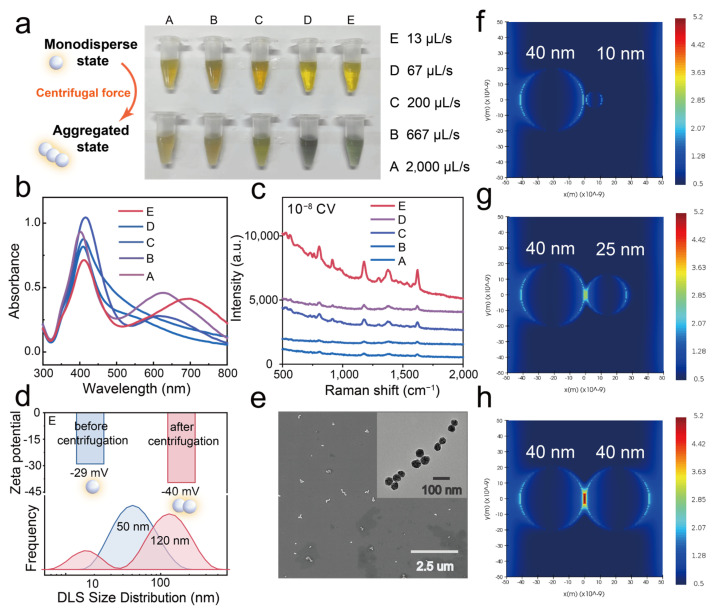
(**a**) Photographs showing the color changes of AgNP sols prepared at different silver nitrate dripping rates before and after centrifugation. (**b**) UV-Vis spectra of AgNP sols after centrifugation. (**c**) SERS spectra of 10^−8^ M crystal violet obtained with different AgNP sols after centrifugation-induced aggregation. (**d**) Zeta potentials and particle size distributions (DLS) of E-AgNPs before and after centrifugation. (**e**) SEM and TEM images of E-AgNPs after centrifugation. (**f**–**h**) FDTD electromagnetic field simulations of different AgNP dimers under 633 nm excitation.

In contrast, our study reveals a distinct centrifugation-induced uniform aggregation phenomenon (Figure S1b). The silver nanoparticles synthesized herein form aggregates at the bottom of the centrifuge tube after centrifugation, and these aggregates can be rapidly and completely redispersed in deionized water. The sol’s color changes markedly, indicating a high degree of aggregation while retaining good dispersibility. This is completely different from the behavior of various traditional ligand-modified silver nanoparticles after centrifugation (Figure S2). After centrifugation at a maximum speed of 13,200 rpm for 15 min, the color and UV-Vis spectra of five different β-CD–stabilized AgNP colloids vary to different extents (Figure 3a). As the silver nitrate addition rate decreases, the centrifugation-induced aggregation becomes more pronounced. Specifically, when the silver colloid is prepared at a silver nitrate addition rate of 13 µL/min (sample E), the nanoparticles change in color from yellow (monodispersed sol) to green (aggregated state), and a new, significantly red-shifted LSPR peak emerges in the 550–750 nm range, indicating strong plasmonic coupling among the aggregated nanoparticles. By comparing the decrease in the LSPR peak at 410 nm in the UV-Vis spectra before (Figure 2c) and after centrifugation (Figure 3b), the aggregation ratio of the five samples can be roughly estimated. Overall, as the silver nitrate addition rate decreases, the nanoparticle size distribution becomes more uniform, and the degree of aggregation increases.

We further evaluated the SERS performance of the five aggregates using 10^−8^ M crystal violet as a model SERS reporter (Figure 3c). As the silver nitrate addition rate decreases, the SERS intensity gradually increases. At an addition rate of 13 µL/min, the SERS intensity reaches its maximum value, so we selected this condition for subsequent applications.

We infer that this improvement is not only attributable to a higher aggregation ratio but also to the enhanced SERS performance of large, uniform silver nanoparticles with wider interparticle gaps. To verify this, we employed three-dimensional finite-difference time-domain (FDTD) simulations to model the electromagnetic field distribution of silver nanoparticles in different aggregation states. We constructed dimer models consisting of one silver nanosphere fixed at 40 nm and a second sphere of either 40 nm, 25 nm, or 10 nm, separated by a constant 2 nm gap, to simulate aggregates formed after centrifugation. As shown in Figure 3f–h, as the size of the second silver nanoparticle increases and approaches 40 nm, the electromagnetic field strength between the two nanoparticles intensifies. This finding suggests that silver nanoparticles with uniform sizes—achieved by controlling the silver nitrate addition rate—generate more SERS hotspots during aggregation, thereby resulting in superior SERS performance. Consequently, we selected the aggregates formed at the slowest silver nitrate addition rate (E) as the optimized colloidal SERS substrate for subsequent experiments.

Furthermore, we evaluated the zeta potential and particle size distribution (measured by dynamic light scattering, DLS) of E-AgNPs before and after centrifugation to investigate in situ changes during the process. Figure 3d shows that unlike aggregates prepared using other chemical methods (e.g., using halide salts), our centrifugation-induced aggregation strategy does not significantly affect the nanoparticles’ surface potential. The zeta potential remains roughly the same (−30 mV) in the synthesis solution before and after centrifugation (Figure S3). Upon redispersion of the silver nanoparticle aggregates (AgNAs) in deionized water, the zeta potential further decreases to −40 mV, indicating improved stability and potential for high reproducibility over extended testing periods. Long-term UV-Vis monitoring demonstrates that the aggregates produced using our centrifugation-induced method remain stable, exhibiting nearly identical spectra for up to 2 h (Figure S4). In contrast, the UV absorption spectra of salt-induced aggregates change significantly within 15 min, indicating that they are markedly unstable. DLS measurements reveal that the particle size increases from 50 nm before centrifugation to 120 nm after centrifugation, accompanied by a narrower size distribution, suggesting that the aggregates formed via centrifugation are more uniform (Figure 3d). It should be noted that a peak near 10 nm appeared in the DLS results after centrifugation, which might be related to the spontaneous aggregation of free β-CD molecules induced by centrifugation [25,26,27,28,29,30]. SEM and TEM images further provide direct observations of the aggregation state (Figure 3e). Interestingly, most aggregates consist of 2–10 individual nanoparticles arranged linearly, a uniform assembly pattern that correlates, to some extent, with the double LSPR peaks observed in the UV-Vis spectrum. We tentatively attribute the aggregation phenomenon during centrifugation to enhanced hydrophobic interactions and hydrogen bonding between β-CD molecules on the surface of the silver nanoparticles [31]. However, the detailed mechanism requires further investigation through experimental studies.

### 3.3. High SERS Enhancement of Centrifugation-Induced Aggregates

**Figure 4:**
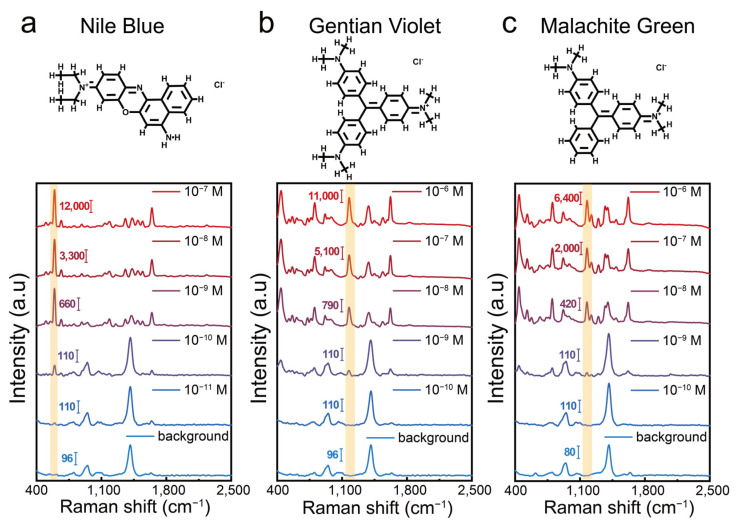
(**a**) Molecular structure of Nile Blue (NB) and corresponding SERS spectra of NB at concentrations from 10^−7^ to 10^−11^ M detected using centrifugation-induced aggregates. (**b**) Molecular structure of Crystal Violet (CV) and corresponding SERS spectra of CV at concentrations from 10^−6^ to 10^−10^ M detected using centrifugation-induced aggregates. (**c**) Molecular structure of Malachite Green (MG) and corresponding SERS spectra of MG at concentrations from 10^−6^ to 10^−10^ M detected using centrifugation-induced aggregates.

In the centrifugation-induced aggregation strategy, the compression of nanoparticles by centrifugal force plays the most direct role in enhancing ligand interactions and triggering nanoparticle aggregation. Thus, key parameters, including the centrifugation speed and time, were optimized in this work. The optimal experimental conditions were found to be 9000 rpm for 15 min (Figure S5). Under these conditions, we tested the SERS enhancement performance of the aggregates using three dye molecules—Nile Blue (NB), Crystal Violet (CV), and Malachite Green (MG)—as model analytes. Using Nile Blue as a representative analyte (Figure 4a), we observed a prominent peak at 593 cm^−1^—corresponding to characteristic C–C–C and C–N–C deformation vibrations—when its concentration reached or exceeded 10^−11^ M. The substrate itself exhibits a relatively simple, weak background signal, featuring a peak at 940 cm^−1^ and another at 1412 cm^−1^. The 940 cm^−1^ peak is likely due to the C–O–C stretching vibrations of glycosidic bonds in β-CD or the C–O stretching of oxidized glucose products, whereas the 1412 cm^−1^ peak may arise from C–H bending in β-CD or from the symmetric stretching of carboxylate groups produced during glucose oxidation. Notably, at Nile Blue concentrations above 10^−9^ M, this minimal background does not significantly interfere with the detection of its characteristic peaks, ensuring clear spectral identification. As shown in Figure 4a–c, the detection limits for NB, CV, and MG were 1 × 10^−11^ M, 1 × 10^−9^ M, and 1 × 10^−9^ M, respectively, demonstrating that the centrifugation-induced aggregates exhibit outstanding SERS performance comparable to or exceeding that of some previously reported colloidal aggregates [32]. These results highlight the remarkable sensitivity of the prepared colloidal SERS substrates, offering a promising approach for the detection of trace analytes.

### 3.4. High SERS Reproducibility

Colloidal SERS substrates are highly favored for their spatial homogeneity and mobility. However, a long-term detection window with high reproducibility is essential for their practical application, particularly in studies that depend on the absolute intensity of SERS signals for quantitative analysis.

**Figure 5:**
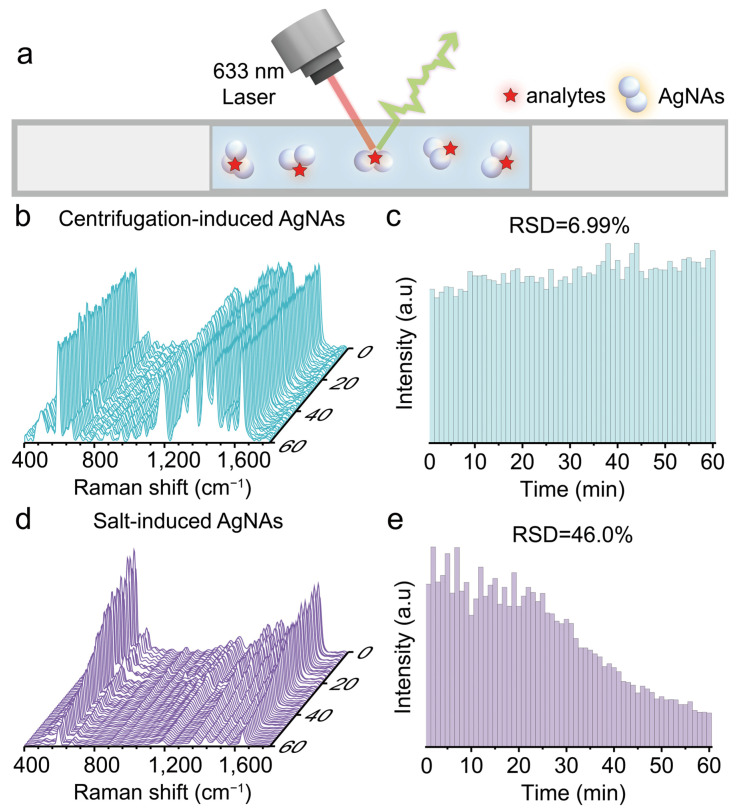
(**a**) Schematic illustration of static SERS detection inside a capillary tube. (**b**) Waterfall plots of continuous static SERS spectra collected within 1 h for 10^−7^ M Nile Blue using centrifugation-induced AgNAs. (**d**) Waterfall plots of continuous static SERS spectra collected within 1 h for 10^−7^ M Nile Blue using 50 mM NaCl-induced AgNAs. (**c**,**e**) Comparison of temporal changes in the intensity of the SERS peak at 595 cm^−1^ for Nile Blue detected using centrifugation-induced and salt-induced AgNAs.

To test the detection window of our colloidal SERS substrate, we measured the reproducibility of SERS signals of centrifugation-induced AgNAs and traditional salt-induced AgNAs over a 1 h period. Equal amounts of both types of aggregates, mixed with the analyte (10^−7^ M NB), were drawn into a glass capillary, and SERS scans were performed every minute throughout the hour (Figure 5a). The relative standard deviation (RSD) of the SERS signal for NB using the centrifugation-induced AgNAs was found to be 6.99% (Figure 5b,c). This excellent reproducibility is attributed to the fact that aggregation induced by centrifugation ceases immediately after the centrifugal force is removed, leaving the colloidal system in a relatively stable and controlled state. In contrast, the RSD of the SERS signals from salt-induced (NaCl) aggregation dramatically increased to 46.0% within 1 h (Figure 5d,e). This poor reproducibility is due to irreversible and uncontrollable aggregation triggered by charge shielding effects from added salt, which leads to continuous aggregation over time and subsequent sedimentation of larger nanoparticle clusters. These findings clearly demonstrate the superior reproducibility and extended detection window of the colloidal SERS substrate produced via centrifugation.

### 3.5. Potential of Continuous High-Throughput SERS Detection

**Figure 6:**
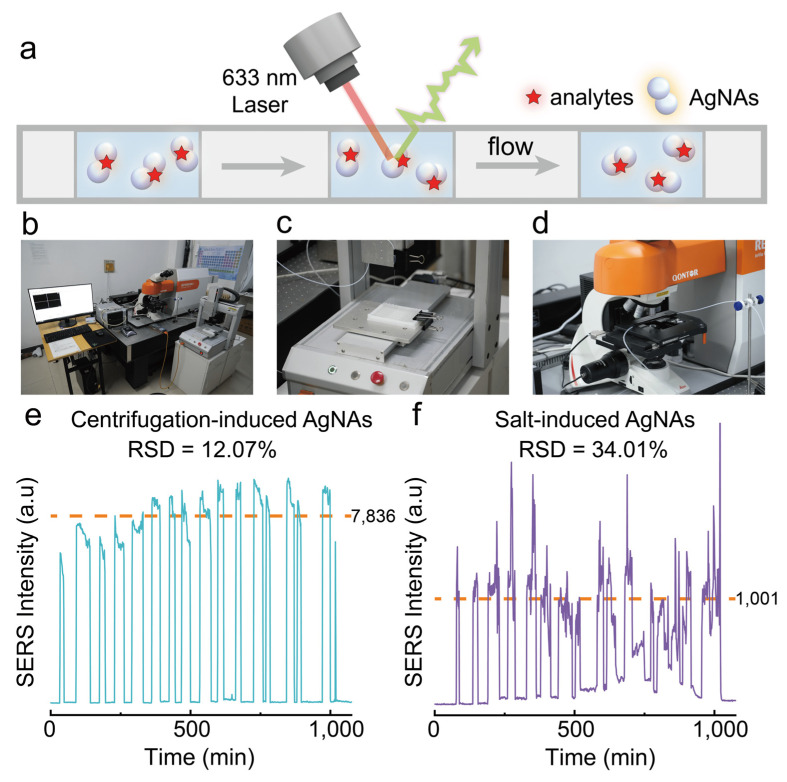
(**a**) Schematic of dynamic high-throughput SERS measurement. (**b**) Self-constructed high-throughput continuous SERS detection platform. (**c**) Continuous sampling module. (**d**) Raman detection module. (**e**,**f**) Reproducibility of high-throughput continuous SERS spectra measurements for 10^−7^ M Nile Blue using centrifugation-induced and salt-induced AgNAs.

Furthermore, we constructed a high-throughput SERS detection platform to evaluate the application potential of centrifugation-induced AgNAs for continuous multi-sample analyses (Figure 6a,b). This platform integrates an automated sampling module (Figure 6c) and a continuous-flow SERS detection module (Figure 6d). Nile Blue (NB) was selected as the target analyte to assess the reproducibility of SERS signals, which were obtained from 15 replicate samples tested sequentially at a constant flow rate as they passed through the laser focal point. As shown in Figure 6e, the RSD of the SERS signals using our centrifugation-induced AgNAs was 12.07%. The relatively high RSD is attributed to the adsorption of β-CD-modified AgNAs on the inner walls of polytetrafluoroethylene tubing during sample flow. Future studies may mitigate this variability through inner-wall surface modification. In comparison, the RSD obtained using conventional salt-induced aggregates (NaCl) was 34.01% (Figure 6f), markedly higher than that of our centrifugation-induced aggregates. Overall, our centrifugation-induced aggregates exhibit exceptional dispersibility and superior reproducibility of SERS signals, demonstrating considerable potential for applications in continuous high-throughput SERS detection.

### 3.6. Detection of Pyocyanin in Real Samples

Pseudomonas aeruginosa is a Gram-negative, waterborne opportunistic pathogen widely present in lakes, seawater, drinking water, and artificial water systems (e.g., storage pipelines and household water filters). The bacterium is highly adaptable, capable of surviving and proliferating in nutrient-deficient distilled water and forming biofilms to resist external stressors. Notably, its substantial resistance to chlorination and alcohol-based disinfectants contributes to its persistent presence in water treatment systems, significantly increasing secondary contamination and infection risks in drinking water. Human exposure to contaminated water may cause symptoms such as diarrhea, vomiting, and severe dehydration, posing particular dangers to immunocompromised individuals.

Pyocyanin, a key toxic metabolite secreted by *P. aeruginosa*, is a redox-active phenazine compound known for inducing host cell damage through reactive oxygen species (ROS) generation. It exhibits dual characteristics of ecological toxicity and pathogenicity. Its environmental concentration directly reflects *P. aeruginosa* biological activity and contamination levels, serving as a sensitive biomarker for assessing pathogen risk in water. Nevertheless, traditional analytical methods, such as high-performance liquid chromatography (HPLC) and microbiological culturing, require complicated sample pretreatment and costly laboratory equipment, limiting their applicability in the real-time monitoring of swimming pools, lakes, or drinking water sources.

**Figure 7:**
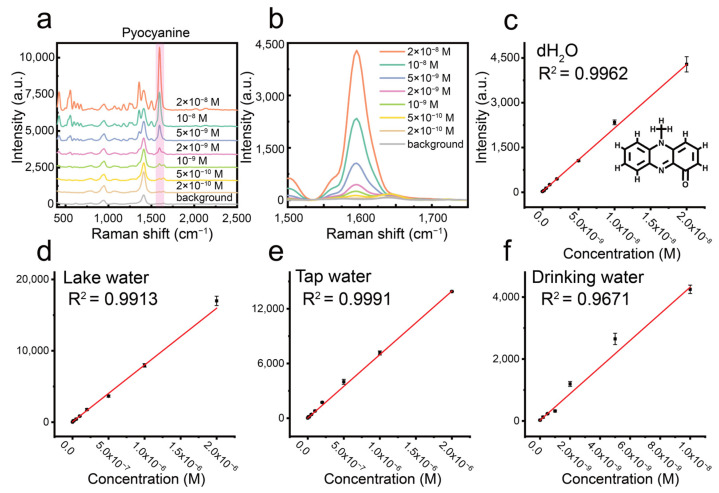
(**a**) SERS spectra of pyocyanin at different concentrations (2 × 10^−8^–2 × 10^−10^ M). (**b**) Magnified view of the SERS peak at 1600 cm^−1^ of pyocyanin at various concentrations. (**c**) Calibration curve of pyocyanin concentration in deionized water. (**d**,**e**) Calibration curves of pyocyanin concentration in lake water and tap water after 50-fold dilution. (**f**) Calibration curve of pyocyanin concentration in drinking water.

To test the practical application of our centrifugation-induced silver nanoparticle aggregates, we developed a SERS-based method for the rapid and sensitive determination of pyocyanin in various water samples. Initially, by optimizing the aggregate concentration (Figure S6), we established an ultra-sensitive SERS protocol for pyocyanin in deionized water, achieving a detection limit of 0.2 nM (Figure 7a,b), a linear response range of 0.5–20 nM (Figure 7c), and excellent linearity (R^2^ = 0.996). The analytical enhancement factor (AEF) for our aggregates with pyocyanin was approximately 3.5 × 10⁶. These results outperform the majority of previously reported SERS methods for pyocyanin detection, as summarized in Table S1, which compares the detection limits from various studies. Furthermore, we evaluated the practical applicability of our method through spike-recovery experiments using drinking water, tap water, and lake water samples (Figure 7d–f). To minimize interference from the complex matrices (especially inorganic salt ions at the millimolar level) in tap and lake water, the samples were diluted 50-fold. Standard curves for each sample type yielded recovery rates ranging from 82% to 117% (Table 1), confirming the robust potential of our centrifugation-induced aggregates for practical environmental water analysis.

## 4. Conclusions

In this work, we demonstrate a centrifugation-induced method for preparing stable colloidal aggregates of β-cyclodextrin-stabilized silver nanoparticles (β-CD@AgNPs). We found that the size uniformity of the silver nanoparticles improved significantly as the silver nitrate addition rate decreased during synthesis. Remarkably, a simple centrifugation step enables the formation of highly dispersed and homogeneous colloidal silver aggregates. Our FDTD simulations further confirm that the SERS sensitivity is strongly dependent on the uniformity of the β-CD@AgNPs. In addition to exhibiting excellent SERS enhancement, these aggregates provide a long detection window compared to conventional salt-induced aggregates and contribute to enhanced SERS stability and reproducibility, which can be attributed to their stable surface charges. The colloidal SERS substrate exhibits outstanding sensitivity to various dye molecules, including Nile Blue, Crystal Violet, and Malachite Green, with detection limits at the nanomolar level. Specifically, using Nile Blue as the target analyte, the aggregates demonstrated satisfactory SERS reproducibility in both static continuous detection (RSD = 6.99%) and automated dynamic sampling (RSD = 12.07%), highlighting their potential for high-throughput continuous analysis. Moreover, when applied to the detection of pyocyanin, a biomarker of Pseudomonas aeruginosa, a detection limit as low as 0.2 nM was achieved, with a linear response range of 0.5–20 nM (R^2^ = 0.996). Spike-recovery experiments in drinking water, tap water, and lake water yielded recoveries between 82% and 117%, demonstrating the practical applicability of the centrifugation-induced aggregates for real sample analysis. This study provides a novel strategy for fabricating stable colloidal SERS substrates and contributes to further improvements in practical SERS analysis.

## Figures and Tables

**Table 1:** Recovery rate of pyocyanin in actual samples.

Samples	Pretreatment	Spiked Concentration (nM)	Detected	Recovery (%)
Drinking water		5.0	5.8	117%
		1.0	8.2	82%
Tap water	Dilute 50 times	100.0	111.4	111%
	Dilute 50 times	50.0	58.6	117%
Lake water	Dilute 50 times	100.0	111.1	111%
	Dilute 50 times	50.0	45.5	91%

## Data Availability

Detailed data can be obtained from the authors.

## Supplementary Materials

The following supporting information can be downloaded at https://www.mdpi.com/article/10.3390/bios15050298/s1: Figure S1. Comparison photographs of different nanoparticles before and after centrifugation under various conditions. Figure S2. Photographs of silver nanoparticles modified with different ligands, before (left) and after centrifugation at 9000 rpm for 15 min, dispersed in ultrapure water (right). Figure S3. Zeta potentials of E-AgNPs (referenced in the main text) before centrifugation, after centrifugation, and following redispersion in both the original synthesis solution and deionized water. Figure S4. (a) UV-Vis absorption spectra of centrifugation-induced AgNAs monitored over 4.5 h. (b) UV-Vis absorption spectra of salt-induced AgNAs monitored over 0.5 h. Figure S5. Optimization of centrifugation speed and duration based on the SERS peak intensity at 595 cm^−1^ for 10^−7^ M Nile Blue. Figure S6. Optimization of AgNA concentration based on the SERS peak intensity at 1600 cm^−1^ for 10^−7^ M Nile Blue. Table S1. Comparison of Detection Limits for pyocyanin in Recent SERS-Based Studies. References [33,34,35,36,37,38,39,40] are cited in the supplementary materials.
