# Supplementary material for: Centrifugation-Induced Stable Colloidal Silver Nanoparticle Aggregates for Reproducible Surface-Enhanced Raman Scattering Detection
Source: Biosensors (Basel). 2025 May 8;15(5):298. doi: 10.3390/bios15050298 (PMC12110687; doi:10.3390/bios15050298)
Supplement: Supplementary file 1 [file biosensors-15-00298-s001.zip › biosensors-3579262-supplementary.pdf]

Centrifugation-Induced Stable Colloidal Silver Nanoparticle Aggregates for Reproducible Surface-Enhanced Raman Scattering Detection

Tianyu Zhou [1,2] and Zhiyang Zhang [1,3,*]

[1] CAS Key Laboratory of Coastal Environmental Processes and Ecological Remediation, Yantai Institute of Coastal Zone Research, Chinese Academy of Sciences, Yantai 264003, China; zhoutianyu22@mails.ucas.ac.cn
[2] University of Chinese Academy of Sciences, Beijing 100049, China
[3] Center for Ocean Mega-Science, Chinese Academy of Sciences, Qingdao 266071, China
* Correspondence: zyzhang@yic.ac.cn

Supplementary Materials

Figures

Figure S1. Comparison photographs of different nanoparticles before and after centrifugation under various conditions.

Figure S2. Photographs of silver nanoparticles modified with different ligands, before (left) and after centrifugation at 9000 rpm for 15 min, dispersed in ultrapure water (right).

Figure S3. Zeta potentials of E-AgNPs (referenced in the main text) before centrifugation, after centrifugation, and following redispersion in both the original synthesis solution and deionized water.

Figure S4. (a) UV-Vis absorption spectra of centrifugation-induced AgNAs monitored over 4.5 h. (b) UV-Vis absorption spectra of salt-induced AgNAs monitored over 0.5 h.

Figure S5. Optimization of centrifugation speed and duration based on the SERS peak intensity at 595 cm^{-1} for 10^{-7} M Nile Blue

Figure S6. Optimization of AgNAs concentration based on the SERS peak intensity at 1600 cm^{-1} for 10^{-7} M pyocyanin.

Table

Table S1. Comparison of Detection Limits for pyocyanin in Recent SERS-Based Studies

Figures

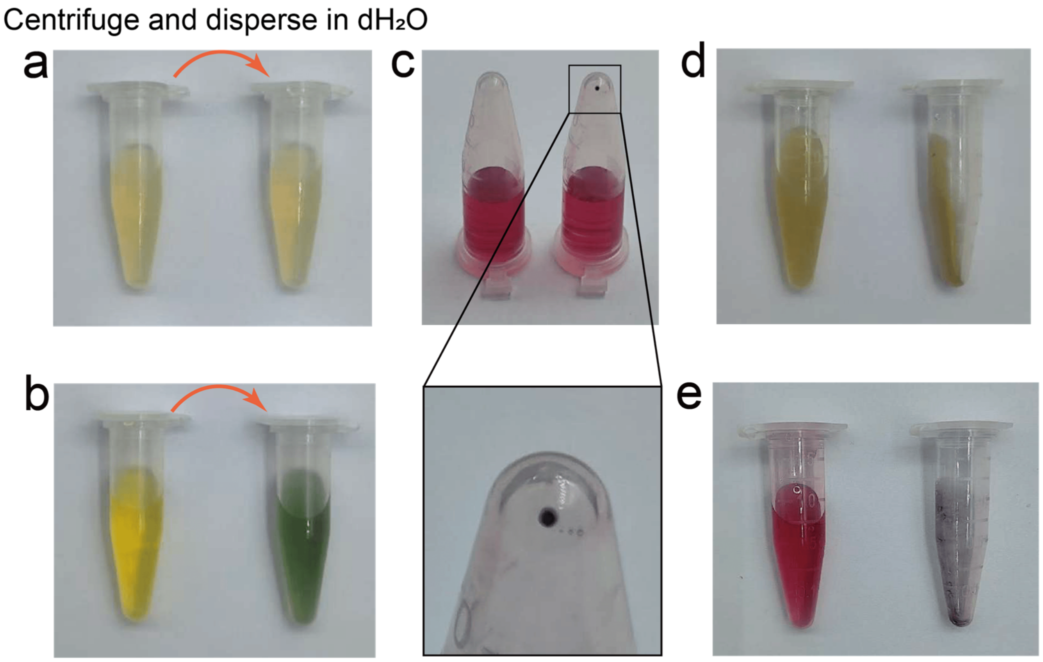

Figure S1. Comparison photographs of different nanoparticles before and after centrifugation under various conditions. (a) Photographs of citrate-stabilized AgNPs before (left) and after (right) centrifugation. (b) Photographs of β-CD-stabilized AgNPs described in the main text before (left) and after (right) centrifugation. (c) Photographs showing the sol color of citrate-stabilized AuNPs before (left) and after (right) centrifugation at 13200 rpm for 15 min. (d) Photographs of citrate-stabilized AgNPs under weakly acidic conditions before (left) and after (right) centrifugation. (e) Photographs of citrate-stabilized AuNPs before (left) and after (right) centrifugation in the presence of 5 mM NaCl.

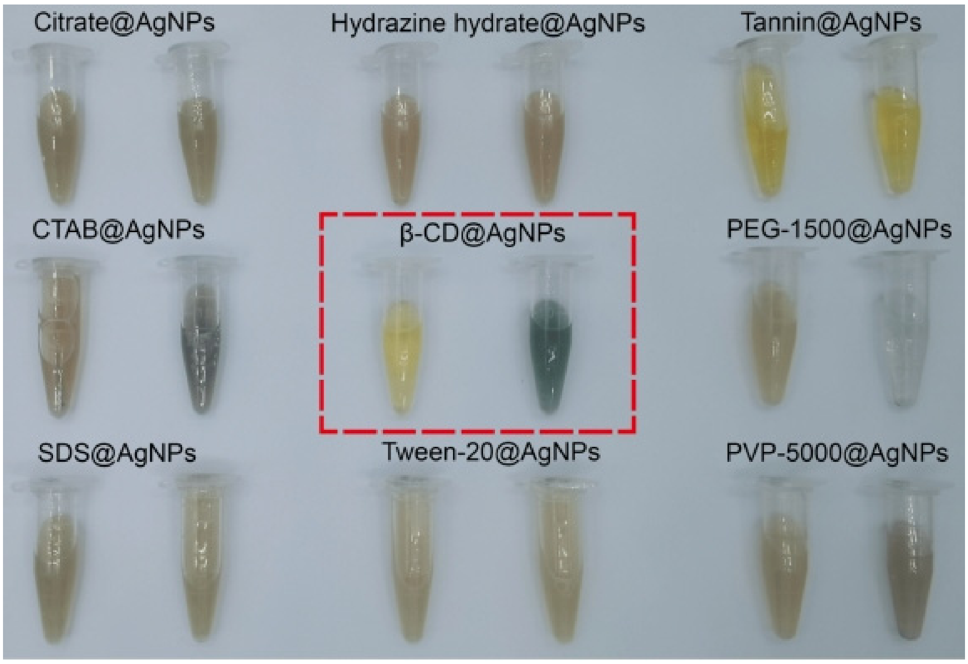

Figure S2. Photographs of silver nanoparticles modified with different ligands, before (left) and after centrifugation at 9000 rpm for 15 min, dispersed in ultrapure water (right).

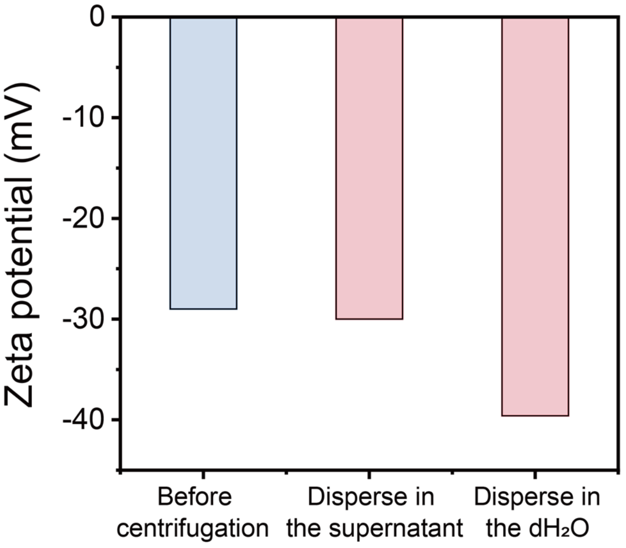

Figure S3. Zeta potentials of E-AgNPs (referenced in the main text) before centrifugation, after centrifugation, and following redispersion in both the original synthesis solution and deionized water.

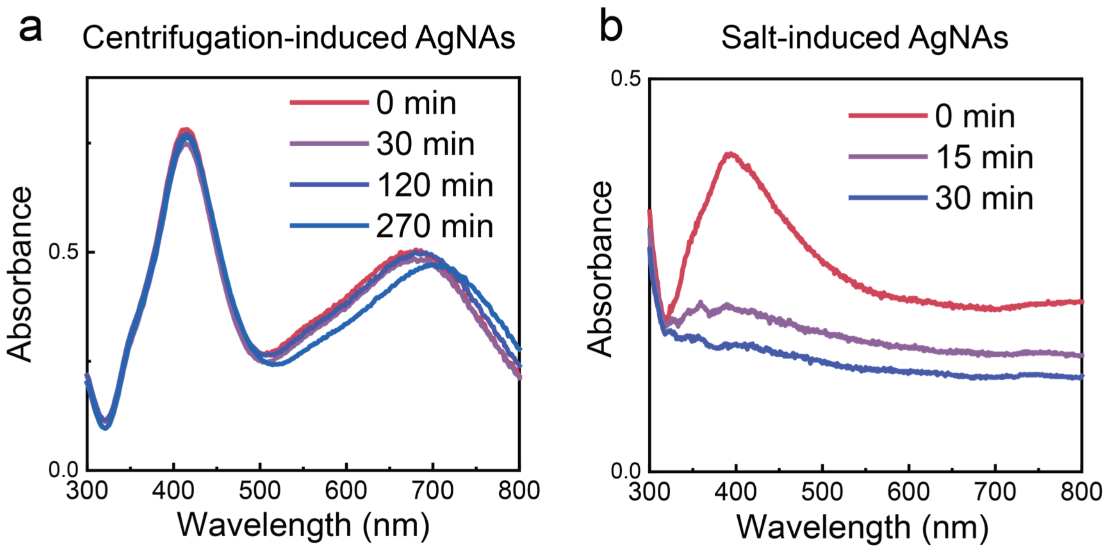

Figure S4. (a) UV-Vis absorption spectra of centrifugation-induced AgNAs monitored over 4.5 h. (b) UV-Vis absorption spectra of salt-induced AgNAs monitored over 0.5 h.

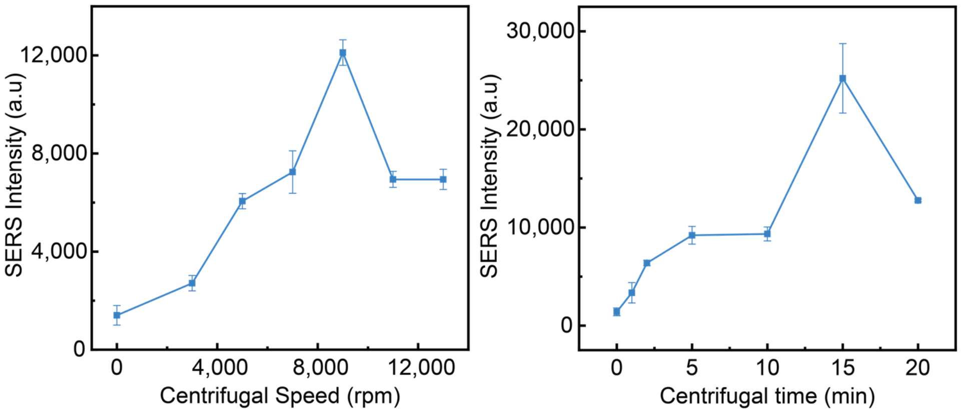

Figure S5. Optimization of centrifugation speed and duration based on the SERS peak intensity at 595 cm^{-1} for 10^{-7} M Nile Blue.

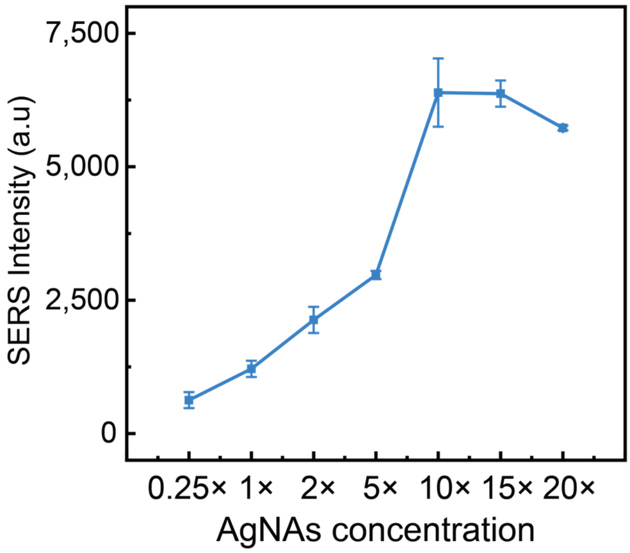

Figure S6. Optimization of AgNAs concentration based on the SERS peak intensity at 1600 cm^{-1} for 10^{-7} M pyocyanin. “1×” denotes the original silver sol concentration immediately after synthesis.

Table

Table S1. Comparison of Detection Limits for pyocyanin in Recent SERS-Based Studies

SERS Substrate	*Years*	*Detection Limit for pyocyanin*	*Reference*
Silver nanorod array	*2014*	2.4×10^{-8} *M*	*[1]*
KCl-induced AgNAs	*2017*	5×10^{-7} *M*	*[2]*
EHD flow induced chemical crosslinking Chemically assembled AuNPs	*2018*	4.8×10^{-10} *M*	*[3]*
Zein/Au-AuNPs	*2019*	1×10^{-8} *M*	*[4]*
AgNPs	*2021*	1.1×10^{-6} *M*	*[5]*
Au electrodeposition onto paper-based 3D SERS substrates	*2022*	5.6×10^{-7} *M*	*[6]*
Sharp-Branched AuNSs	*2023*	5×10^{-11} *M*	*[7]*
Rough Au/Alumina Platforms	*2024*	9.6×10^{-8} *M*	*[8]*
Centrifugation-induced AgNAs		2×10^{-10} *M*	*This work*

References

1. Wu, X.; Chen, J.; Li, X.; Zhao, Y.; Zughaier, S.M. Culture-free diagnostics of Pseudomonas aeruginosa infection by silver nanorod array based SERS from clinical sputum samples. *Nanomedicine: Nanotechnology, Biology and Medicine* **2014**, *10*, 1863-1870, doi:https://doi.org/10.1016/j.nano.2014.04.010.
2. Žukovskaja, O.; Jahn, I.J.; Weber, K.; Cialla-May, D.; Popp, J. Detection of Pseudomonas aeruginosa Metabolite Pyocyanin in Water and Saliva by Employing the SERS Technique. *Sensors* **2017**, *17*, 1704.
3. Nguyen, C.Q.; Thrift, W.J.; Bhattacharjee, A.; Ranjbar, S.; Gallagher, T.; Darvishzadeh-Varcheie, M.; Sanderson, R.N.; Capolino, F.; Whiteson, K.; Baldi, P.; et al. Longitudinal Monitoring of Biofilm Formation via Robust Surface-Enhanced Raman Scattering Quantification of Pseudomonas aeruginosa-Produced Metabolites. *ACS Applied Materials & Interfaces* **2018**, *10*, 12364-12373, doi:10.1021/acsami.7b18592.
4. Jia, F.; Barber, E.; Turasan, H.; Seo, S.; Dai, R.; Liu, L.; Li, X.; Bhunia, A.K.; Kokini, J.L. Detection of Pyocyanin Using a New Biodegradable SERS Biosensor Fabricated Using Gold Coated Zein Nanostructures Further Decorated with Gold Nanoparticles. *J. Agric. Food Chem.* **2019**, *67*, 4603-4610, doi:10.1021/acs.jafc.8b07317.

5. Tanaka, Y.; Khoo, E.H.; Salleh, N.A.b.M.; Teo, S.L.; Ow, S.Y.; Sutarlie, L.; Su, X. A portable SERS sensor for pyocyanin detection in simulated wound fluid and through swab sampling. *Analyst* **2021**, *146*, 6924-6934, doi:10.1039/D1AN01360B.
6. Kim, S.; Ansah, I.B.; Park, J.S.; Dang, H.; Choi, N.; Lee, W.-C.; Lee, S.H.; Jung, H.S.; Kim, D.-H.; Yoo, S.M.; et al. Early and direct detection of bacterial signaling molecules through one-pot Au electrodeposition onto paper-based 3D SERS substrates. *Sensors Actuators B: Chem.* **2022**, *358*, 131504, doi:https://doi.org/10.1016/j.snb.2022.131504.
7. Atta, S.; Vo-Dinh, T. Solution-Based Ultra-Sensitive Surface-Enhanced Raman Scattering Detection of the Toxin Bacterial Biomarker Pyocyanin in Biological Fluids Using Sharp-Branched Gold Nanostars. *Anal. Chem.* **2023**, *95*, 2690-2697, doi:10.1021/acs.analchem.2c03210.
8. El-Said, W.A.; Saleh, T.S.; Al-Bogami, A.S.; Wani, M.Y.; Choi, J.-w. Development of Novel Surface-Enhanced Raman Spectroscopy-Based Biosensors by Controlling the Roughness of Gold/Alumina Platforms for Highly Sensitive Detection of Pyocyanin Secreted from Pseudomonas aeruginosa. *Biosensors* **2024**, *14*, 399.
